# The genome sequence of the brown trout, *Salmo trutta* Linnaeus 1758

**DOI:** 10.12688/wellcomeopenres.16838.1

**Published:** 2021-05-13

**Authors:** Tom Hansen, Per Gunnar Fjelldal, Sigbjørn Lien, Michelle Smith, Craig Corton, Karen Oliver, Jason Skelton, Emma Betteridge, Jale Doulcan, Olivier Fedrigo, Jacquelyn Mountcastle, Erich Jarvis, Shane A. McCarthy, William Chow, Kerstin Howe, James Torrance, Jonathan Wood, Ying Sims, Leanne Haggerty, Richard Challis, Jonathan Threlfall, Daniel Mead, Richard Durbin, Mark Blaxter

**Affiliations:** 1 Institute of Marine Research (IMR), Matredal, Norway; 2 Norwegian University of Life Sciences, Ås, 1432, Norway; 3 Wellcome Sanger Institute, Wellcome Genome Campus, Hinxton, Cambridge, CB10 1SA, UK; 4 Achilles Therapeutics plc, London, W6 8PW, UK; 5 The Rockefeller University, New York, New York, 10065, USA; 6 Howard Hughes Medical Institute, Chevy Chase, Maryland, 20815, USA; 7 Department of Genetics, University of Cambridge, Cambridge, CB2 3EH, UK; 8 EMBL-EBI, Wellcome Genome Campus, Hinxton, Cambridge, CB10 1SA, UK; 9 Owlstone Medical, Cambridge Science Park, Cambridge, CB4 0GJ, UK

**Keywords:** Salmo trutta, brown trout, genome sequence, chromosomal

## Abstract

We present a genome assembly from an individual female *Salmo trutta *(the brown trout; Chordata; Actinopteri; Salmoniformes; Salmonidae). The genome sequence is 2.37 gigabases in span. The majority of the assembly is scaffolded into 40 chromosomal pseudomolecules. Gene annotation of this assembly on Ensembl has identified 43,935 protein coding genes.

## Species taxonomy

Metazoa; Chordata; Craniata; Actinopterygii; Actinopteri; Neopterygii; Teleostei; Euteleosteomorpha; Salmoniformes; Salmonidae; Salmoninae; Salmo; *Salmo trutta* Linnaeus 1758 (NCBItxid:8032).

## Introduction

The brown trout, *Salmo trutta*, is native to Europe, western Asia and North Africa; however, the species has been successfully introduced to a multitude of other geographical locations ( Klemetsen *et al.*, 2003). Genetically similar *S. trutta* can be freshwater residents, freshwater migrants or anadromous (migrating to the sea to feed, only returning to freshwater to breed), leading taxonomists initially to believe that these were multiple independent species. This phenotypic difference has a genetic component but is also partly caused by environmental factors, such as food availability, which lead to changes in gene expression and drives migration and adaptation to different environments ( Ferguson *et al.*, 2019). *S. trutta* also exhibit considerable genetic variation within migratory or resident populations; these differences can be seen by populations in different habitats ( Ferguson, 1989) or in the same habitat ( Andersson *et al.*, 2017). This genetic diversity can allow populations to occupy different environments, such as those with high levels of acidity ( Prodöhl *et al.*, 2019).

This reference genome sequence will be of utility for researchers that wish to sample and analyse the genetics of *S. trutta* populations, helping to understand genetic drivers behind migration and the reasons why different populations of brown trout are so well adapted to different conditions. As increases in atmospheric CO _2_ continue to increase temperatures and acidify oceans, this information will help conservation of *S. trutta* and other species by revealing which genetic components allow populations to adapt to warmer and more acidic environments.

## Genome sequence report

**Figure 1.**
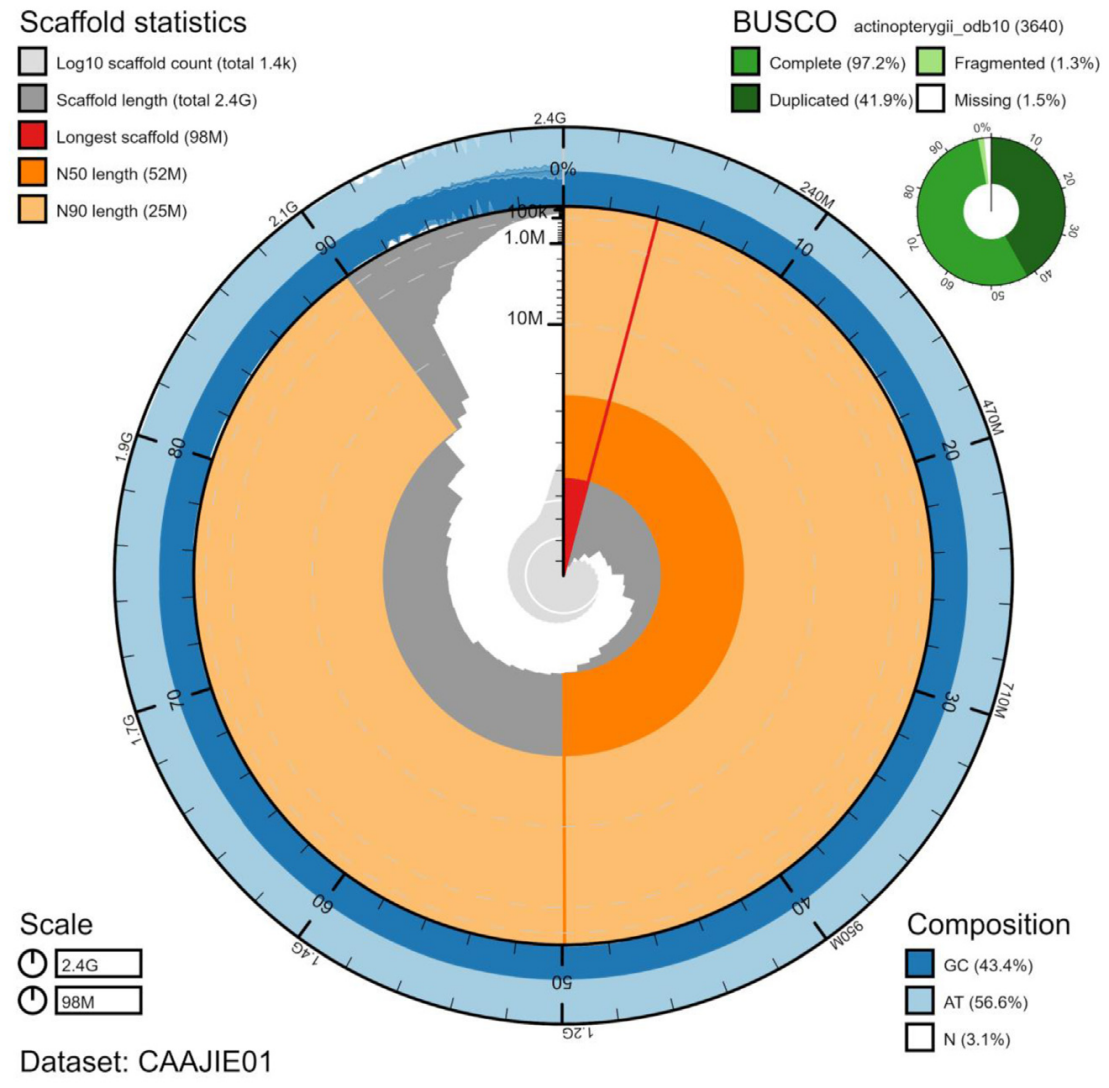
Genome assembly of *Salmo trutta*, fSalTru1.1: metrics. The BlobToolKit Snailplot shows N50 metrics and BUSCO gene completeness. An interactive version of this figure is available at https://blobtoolkit.genomehubs.org/view/Salmo%20trutta/dataset/CAAJIE01/snail.

The genome was sequenced from a single female *Salmo trutta* bred at the Institute of Marine Research, Bergen, Norway. A total of 52-fold coverage in Pacific Biosciences single-molecule long reads (N50 19 kb) and 70-fold coverage in 10X Genomics read clouds (from molecules with an estimated N50 of 65 kb) were generated. Primary assembly contigs were scaffolded with chromosome conformation Hi-C data, and 67-fold coverage of Bionano optical maps. Manual assembly curation corrected 175 missing/misjoins, reducing the scaffold number by 4.8% and the assembly length by 0.5%. The final assembly has a total length of 2.37 Gb in 1,441 sequence scaffolds with a scaffold N50 of 52.21 Mb ( Table 1). The majority, 91.5%, of the assembly sequence was assigned to 40 chromosomal-level scaffolds, representing 40 autosomes (numbered by sequence length). No sex chromosomes could be identified ( Figure 1; Table 2). The assembly has a BUSCO ( Simão *et al.*, 2015) completeness of 97.2% using the actinopterygii_odb10 reference set. Genome assembly metrics, GC coverage, cumulative sequence and the Hi-C contact map are visualised in Figure 1– Figure 4, respectively.

**Table 1.** Genome data for *Salmo trutta*, fSalTru1.1.

*Project accession data*	
Assembly identifier	fSalTru1.1
Species	*Salmo trutta*
Specimen	fSalTru1
NCBI taxonomy ID	txid8032
BioProject	PRJEB32115
BioSample ID	SAMEA994732
Isolate information	Female, muscle
*Raw data accessions*	
PacificBiosciences SEQUEL I	ERX3245920, ERX3253848- ERX3253850, ERX3279922- ERX3279929, ERX3288373, ERX3311049-ERX3311054, ERX3311066, ERX3318044- ERX3318049, ERX3338928, ERX3338929
10X Genomics Illumina	ERX3341615-ERX3341622
Hi-C Illumina	ERX4142808-ERX4142812
BioNano	ERZ1395486
*Genome assembly*	
Assembly accession	GCA_901001165.1
Span (Mb)	2,372
Number of contigs	5,378
Contig N50 length (Mb)	1.7
Number of scaffolds	1441
Scaffold N50 length (Mb)	52.2
Longest scaffold (Mb)	81.5
BUSCO * genome score	C:94.7%[S:49.4%,D:45.3%],F:1.8%, M:3.5%,n:4584
*Genome annotation*	
Number of protein-coding genes	43,935
Average coding sequence length (bp)	2,058
Average number of exons per gene	13
Average exon size (bp)	210
Average intron size (bp)	2,770

*BUSCO scores based on the actinopterygii_odb10 BUSCO set using v5.0.0. C= complete [S= single copy, D=duplicated], F=fragmented, M=missing, n=number of orthologues in comparison. A full set of BUSCO scores is available at https://blobtoolkit.genomehubs.org/view/Salmo%20trutta/dataset/CAAJIE01/busco.

**Table 2.** Chromosomal pseudomolecules in the genome assembly of *Salmo trutta*, fSalTru1.1.

INSDC accession	Chromosome	Size (Mb)	GC%
LR584410.1	1	81.54	43.8
LR584445.1	2	75.35	43.6
LR584416.1	3	74.75	43.6
LR584420.1	4	73.17	43.2
LR584433.1	5	67.76	43.1
LR584406.1	6	60.1	43.5
LR584430.1	7	59.84	43.1
LR584407.1	8	51.19	43.8
LR584409.1	9	49.36	43.5
LR584419.1	10	46.6	43.2
LR584438.1	11	22.96	43.8
LR584441.1	12	97.53	43.8
LR584428.1	13	91.49	43.9
LR584411.1	14	86.25	43.3
LR584415.1	15	66.9	42.9
LR584431.1	16	61.35	43.1
LR584426.1	17	59.76	43.1
LR584435.1	18	59.14	43.1
LR584427.1	19	56.58	43.2
LR584429.1	20	55.16	43.2
LR584437.1	21	52.73	43.4
LR584440.1	22	52.21	43.6
LR584421.1	23	51.49	43.5
LR584412.1	24	50.33	43.2
LR584436.1	25	48.97	43.6
LR584439.1	26	48.7	44
LR584424.1	27	46.41	43.4
LR584422.1	28	46.38	43.5
LR584418.1	29	46.06	43.7
LR584432.1	30	45.79	43.7
LR584423.1	31	45.59	43.1
LR584408.1	32	44.95	43.9
LR584414.1	33	44.89	43.5
LR584434.1	34	42.9	43.9
LR584444.1	35	41.92	43.5
LR584442.1	36	41.68	43.9
LR584417.1	37	35.21	43.8
LR584425.1	38	34.89	43.3
LR584413.1	39	25.83	43.6
LR584443.1	40	25.48	44.1

**Figure 2.**
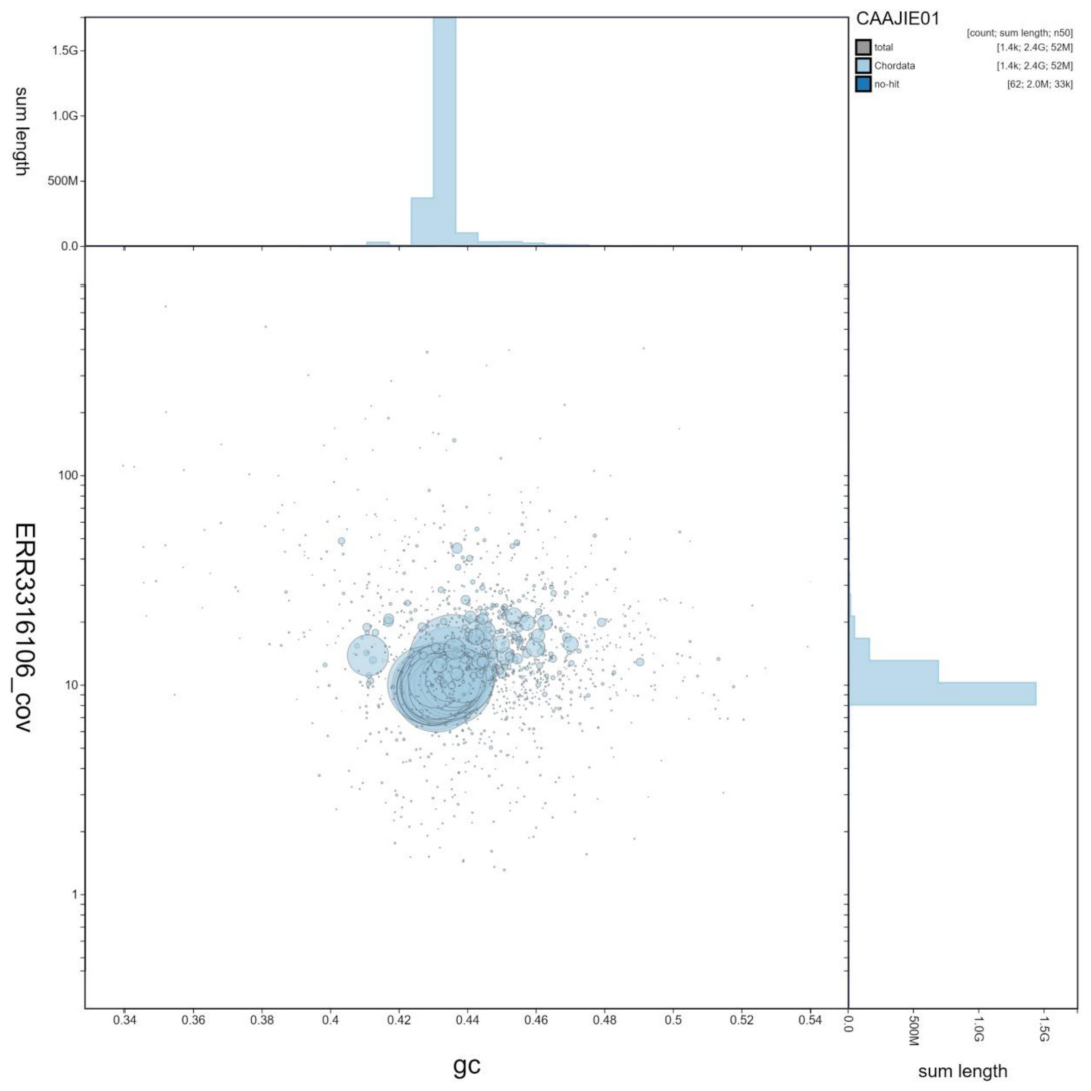
Genome assembly of *Salmo trutta*, fSalTru1.1: GC coverage. BlobToolKit GC-coverage plot. An interactive version of this figure is available at https://blobtoolkit.genomehubs.org/view/Salmo%20trutta/dataset/CAAJIE01/blob?plotShape=circle.

**Figure 3.**
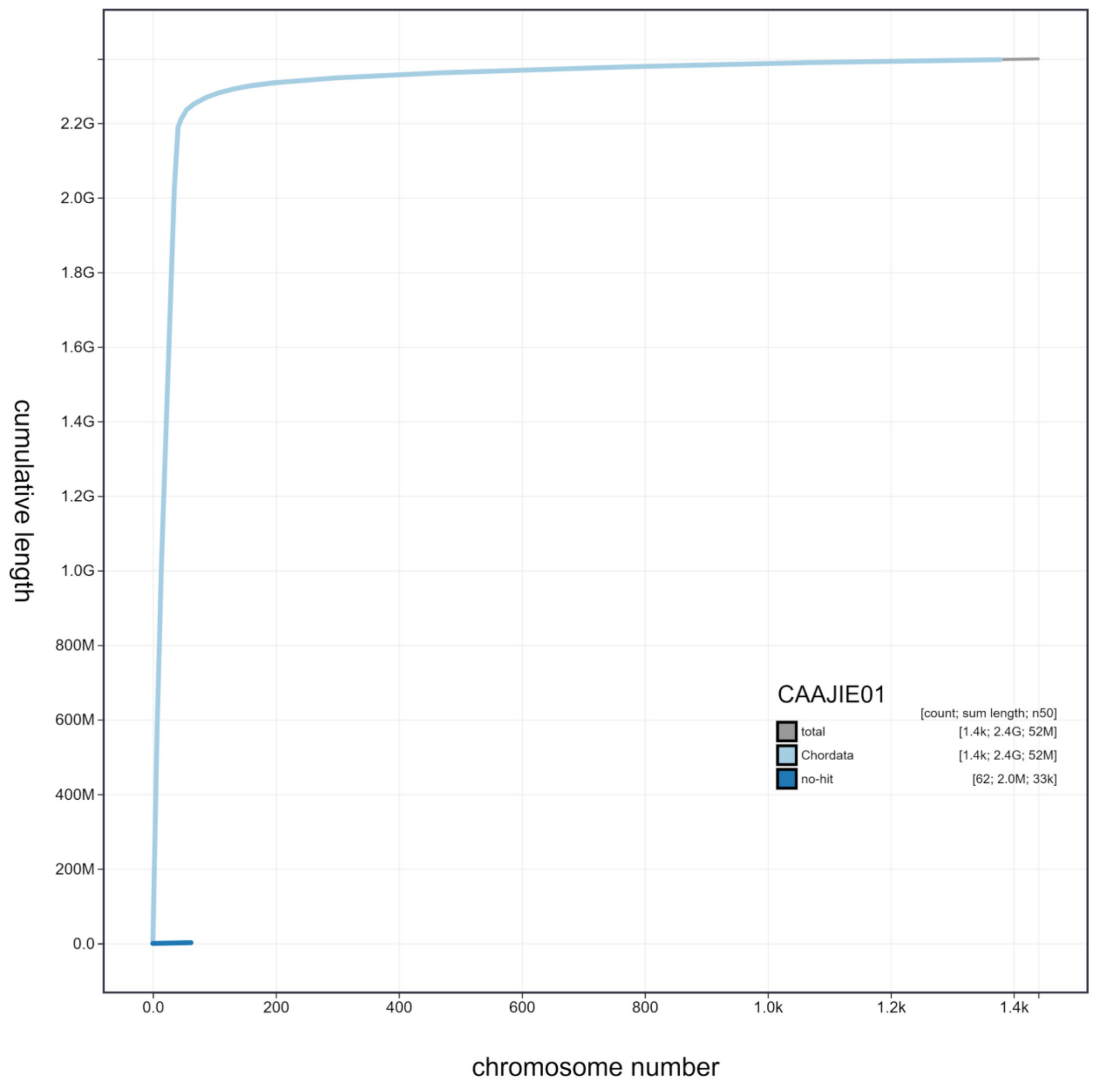
Genome assembly of *Salmo trutta*, fSalTru1.1: cumulative sequence. BlobToolKit cumulative sequence plot. An interactive version of this figure is available at https://blobtoolkit.genomehubs.org/view/Salmo%20trutta/dataset/CAAJIE01/cumulative.

**Figure 4.**
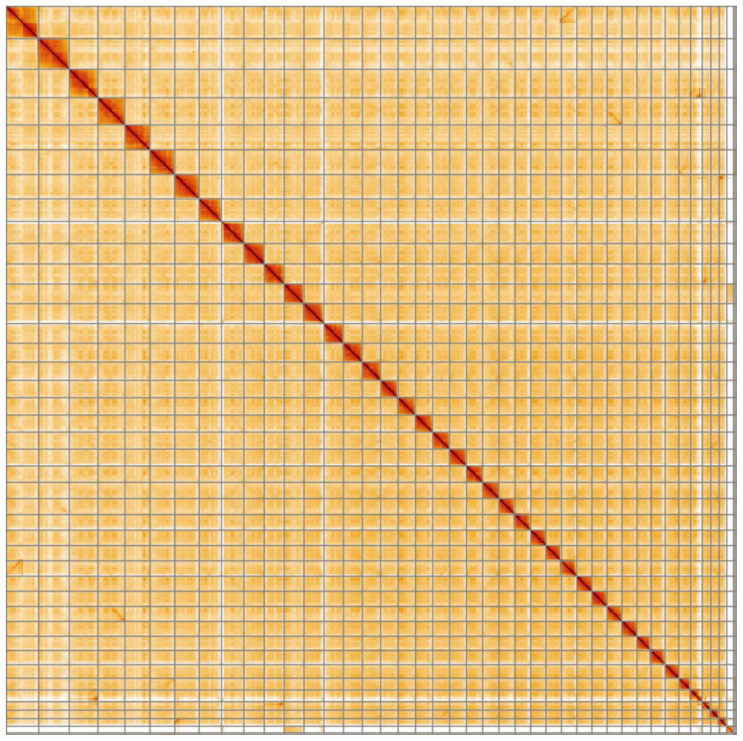
Genome assembly of *Salmo trutta*, fSalTru1.1: Hi-C contact map. Hi-C contact map of the fSalTru1.1 assembly, visualised in HiGlass.

## Gene annotation

The Ensembl gene annotation system ( Aken *et al.*, 2016) was used to generate annotation for the fSalTru1.1 assembly ( GCA_901001165.1) ( Table 1). Annotation was created primarily through alignment of transcriptomic data to the genome, with gap filling via protein-to-genome alignments of a select set of vertebrate proteins from UniProt ( UniProt Consortium, 2019). The resulting Ensembl annotation includes 122,381 transcripts assigned to 43,935 coding and 4,441 non-coding genes ( Salmo trutta - Ensembl Rapid Release).

## Methods

Owing to the high genetic diversity of brown trout and the variable chromosome numbers ( *S. trutta* have 38-42 chromosomes, with multiple copies of these chromosomes), doubled haploid specimens were bred for sequencing and generation of the assembly. The doubled haploid female used in this study was bred on 26 November 2015 at the Institute of Marine Research using a protocol optimized for Atlantic salmon, *Salmo salar* (see ( Hansen *et al.*, 2020)). In summary, eggs from one *Salmo trutta* female from a domestic stock that originated from Lake Tunhovd in eastern Norway were fertilized with UV irradiated milt (brown trout sperm diluted 1:40 with sperm fluid and irradiated (254 nm) for 8 mins at 0.48 mWcm ^2^, activated and left to hydrate in 8°C freshwater in a polyethylene (PE) container. After 4700 min.°C irradiation, the PE bottle was transferred to a pressure chamber and the eggs were subjected to a hydrostatic pressure of 655 bar for 5 mins. The eggs were incubated at approximately 6°C and surviving larvae were fed at 12°C and continuous light until June 2016 when temperature and photoperiod was changed to ambient conditions. On 16 January 2018, one female individual was euthanized (500 mgL− 1 Finquel® (MS 222) and sampled.

The specimen was transferred to the Wellcome Sanger Institute and DNA was extracted using an agarose plug extraction from spleen tissue following the Bionano Prep Animal Tissue DNA Isolation Soft Tissue Protocol.

Sequencing was performed by the Scientific Operations core at the Wellcome Sanger Institute on Pacific Biosciences SEQUEL I and Illumina HiSeq X instruments. Hi-C data were generated using the Arima Hi-C kit v1 by Arima Genomics, San Diego, USA, and sequenced on Illumina HiSeqX. BioNano data were generated in the Rockefeller University Vertebrate Genome laboratory using the Saphyr instrument. Ultra-high molecular weight DNA was extracted using the Bionano Prep Animal Tissue BioNano data were generated in the Rockefeller University Vertebrate Genome laboratory using the Saphyr instrument. Ultra-high molecular weight DNA was extracted using the Bionano Prep Animal Tissue DNA Isolation FibrousTissue Protocol and assessed by pulsed field gel and Qubit 3 fluorimetry. DNA was labeled for Bionano Genomics optical mapping following the Bionano Prep Direct Label and Stain (DLS) Protocol and run on one Saphyr instrument chip flowcell. The total yield of tagged molecules ≥ 150 kb with at least 9 sites was 272.3 Gb (N50 0.28 Mb). A CMAP (Bionano assembly consensus genome map) was *de-novo* assembled using Bionano Solve (see Table 3 for software versions and sources) a total map length of 2.62 Gb and a map N50 of 29.37 Mb.

**Table 3.** Software tools used.

Software tool	Version	Source
Falcon-unzip	falcon-kit 1.2.1	( Chin *et al.*, 2016)
SALSA2	2.1	( Ghurye *et al.*, 2019)
scaff10x	3.0	https://github.com/wtsi-hpag/Scaff10X
arrow	GenomicConsensus 2.2.2	https://github.com/PacificBiosciences/GenomicConsensus
longranger align	2.2.2	https://support.10xgenomics.com/genome-exome/software/ pipelines/latest/advanced/other-pipelines
freebayes	1.1.0-3-g961e5f3	( Garrison & Marth, 2012)
bcftools consensus	1.9	http://samtools.github.io/bcftools/bcftools.html
Bionano Solve	3.2.2_08222018	https://bionanogenomics.com/downloads/bionano-solve/
HiGlass	1.11.6	( Kerpedjiev *et al.*, 2018)
PretextViewer	0.0.4	https://github.com/wtsi-hpag/PretextView
gEVAL	N/A	( Chow *et al.*, 2016)
BlobToolKit	1.2	( Challis *et al.*, 2020)

Assembly was carried out following the Vertebrate Genome Project pipeline v1.0 ( Rhie *et al.*, 2020) with Falcon-unzip ( Chin *et al.*, 2016) and a first round of scaffolding carried out with 10X Genomics read clouds using scaff10x. Hybrid scaffolding was performed using the BioNano DLE-1 data and BioNano Solve. Scaffolding with Hi-C data ( Rao *et al.*, 2014) was carried out with SALSA2 ( Ghurye *et al.*, 2019). The Hi-C scaffolded assembly was polished with arrow using the PacBio data, then polished with the 10X Genomics Illumina data by aligning to the assembly with longranger align, calling variants with freebayes ( Garrison & Marth, 2012) and applying homozygous non-reference edits using bcftools consensus. Two rounds of the Illumina polishing were applied. The assembly was checked for contamination and corrected. Manual curation was performed as described previously ( Howe *et al.*, 2021) using the gEVAL system ( Chow *et al.*, 2016), Bionano Access, HiGlass and Pretext. Figure 1– Figure 3 and BUSCO values were generated using BlobToolKit ( Challis *et al.*, 2020).

## Data availability

### Underlying data

BioProject: Salmo trutta RefSeq Genome, Accession number PRJNA550988: https://www.ncbi.nlm.nih.gov/bioproject/550988

The genome sequence is released openly for reuse. The *S. trutta* genome sequencing initiative is part of the Darwin Tree of Life (DToL) project and the Vertebrate Genome Project (VGP) ordinal references programme. All raw data and the assembly have been deposited in INSDC databases. Raw data and assembly accession identifiers are reported in Table 1.
