# Supplementary material for: The genome sequence of the brown trout, Salmo trutta Linnaeus 1758
Source: Wellcome Open Res. 2021 May 13;6:108. [Version 1] doi: 10.12688/wellcomeopenres.16838.1 (PMC8488904; doi:10.12688/wellcomeopenres.16838.1)
Supplement: Peer review history

# Peer review: Reviewer response for version 1

**DOI:** 10.21956/wellcomeopenres.18573.r46064

**Published:** 2021-10-14

**Authors:** Sajad Nazari

**Affiliations:** 1 Shahid Motahary Cold-Water Fishes Genetic and Breeding Research Center, Iranian Fisheries Sciences Research Institute, Agricultural Research, Education and Extension Organization (AREEO), Yasouj, Iran

The brown trout, *Salmo trutta*, is native to Europe, western Asia and North Africa and is an important fish across these regions. The authors improved the reference genome of *Salmo trutta* using PacBio, Hi-C sequencing technologies which means a much more complete chromosome-level assembly can be feasibly obtained.

 Based on the new assembly, genome analysis was then performed on a female individual.

 The manuscript did a great job demonstrating successful high-quality chromosome level analysis in a non-model species. In particular, the Introduction provides an excellent backdrop to the findings of the paper. Some figures are clear and concise, and the analyses are sufficiently well described in the methods to enable the reader to fully understand what was done.

 In general, the manuscript was clearly written and the analytical methods were sound. I have only a few minor concerns about the paper.

1. Sex determination should be an extremely simple trait. Is it due to genetics or assembly error?
2. There are quite a few inconsistencies between the genetic map and the assembly. Careful checking is needed to make sure the inconsistencies are not due to assembly errors
3. The BUSCO is outdated version. Please consider using BUSCO (Manni *et al.*, 2021). ^1^

Are sufficient details of methods and materials provided to allow replication by others?

Yes

Is the rationale for creating the dataset(s) clearly described?

Yes

Are the datasets clearly presented in a useable and accessible format?

Yes

Are the protocols appropriate and is the work technically sound?

Yes

Reviewer Expertise:

Analysis of the next-generation DNA sequence data, genome assembly, genome annotation

I confirm that I have read this submission and believe that I have an appropriate level of expertise to confirm that it is of an acceptable scientific standard.

## References

[1] : BUSCO Update: Novel and Streamlined Workflows along with Broader and Deeper Phylogenetic Coverage for Scoring of Eukaryotic, Prokaryotic, and Viral Genomes. *Mol Biol Evol* .2021;38(10) : 10.1093/molbev/msab199 4647-4654 10.1093/molbev/msab199 34320186PMC8476166

# Peer review: Reviewer response for version 1

**DOI:** 10.21956/wellcomeopenres.18573.r46069

**Published:** 2021-10-01

**Authors:** Manuel Vera

**Affiliations:** 1 Department of Zoology, Genetics and Physical Anthropology, ACUIGEN group, Faculty of Veterinary, Universidade de Santiago de Compostela, Lugo, Spain

The present data note represents the reference genome sequence of the brown trout, *Salmo trutta*. The population/conservation genetics of this species has been widely studied for the last four decades. The next generation sequencing (NGS) techniques together with Genotyping-by-Sequencing (GBS) methodologies (e.g. RADseq) have allowed the studies of population genomics in the species, but its reference genome was necessary. Thus, this genomic resource, with a very high quality, represents a valuable tool for geneticists interested in *S. trutta *and other salmonids. The interest and workflow (including the protocols) of the manuscript are well presented. Methodology is well described and detailed, allowing their reproducibility by other researchers. Finally, all the data is freely available with useful links (some of them interactive) which facilitates its use and increases the interest of the manuscript.

 I have just a few comments to the authors:

- Page 3, “Introduction” section: Replace “…(Ferguson *et al.,* 2019). *S. trutta* also exhibit…” by “…(Ferguson *et al.,* 2019). *Salmo trutta* also exhibit…”
- Page 3, “Genome sequence report” section: Authors stated that “The majority, 91.5%, of the assembly sequence was assigned to 40 chromosomal-level scaffolds, representing 40 autosomes (numbered by sequence length)”. However, chromosome names shown on Table 2 are not numbered by size (e.g. The length of chromosome 12 (97.53 Mb) is longer than those shown for chromosomes 1-11. Do the authors have information about the relationship among their chromosomal pseudomolecules and the physical chromosomes?
- Page 6, second column: Replace “…was extracted using the Bionano Prep Animal Tissue BioNano data were generated…” by “…was extracted using the Bionano Prep Animal Tissue. BioNano data were generated…” (a dot between the two sentences is missed).

Are sufficient details of methods and materials provided to allow replication by others?

Yes

Is the rationale for creating the dataset(s) clearly described?

Yes

Are the datasets clearly presented in a useable and accessible format?

Yes

Are the protocols appropriate and is the work technically sound?

Yes

Reviewer Expertise:

Population/Conservation genetics of aquatic organisms

I confirm that I have read this submission and believe that I have an appropriate level of expertise to confirm that it is of an acceptable scientific standard.

# Peer review: Reviewer response for version 1

**DOI:** 10.21956/wellcomeopenres.18573.r43865

**Published:** 2021-06-01

**Authors:** Peng Xu

**Affiliations:** 1 College of Fishery, Henan Normal University, Xinxiang, China

This Data Note reported a high-quality chromosome level *Salmo trutta* genome used PacBio, Hi-C and Biono optical maps. The quality of *Salmo trutta* genome is very sound and it will be helpful in the analysis of the genetics of *S. trutta *populations. In this manuscript, the rationale for creating the dataset(s) was described clearly. The protocols and work are technically appropriate. The details of the method and materials are sufficient to allow replication by others. The data links were clearly presented in a useable and accessible format.

 I have only one suggestion as follows:

- In the section of “Genome sequence report” Line 8, please explain the method that you identify the sex chromosome?

Are sufficient details of methods and materials provided to allow replication by others?

Yes

Is the rationale for creating the dataset(s) clearly described?

Yes

Are the datasets clearly presented in a useable and accessible format?

Yes

Are the protocols appropriate and is the work technically sound?

Yes

Reviewer Expertise:

Fish genomics and genetics

I confirm that I have read this submission and believe that I have an appropriate level of expertise to confirm that it is of an acceptable scientific standard.
